# Supplementary material for: Identification of Prognostic Candidate Genes in Breast Cancer by Integrated Bioinformatic Analysis
Source: J Clin Med. 2019 Aug 2;8(8):1160. doi: 10.3390/jcm8081160 (PMC6723760; doi:10.3390/jcm8081160)
Supplement: Supplementary file 1 [file jcm-08-01160-s001.pdf]

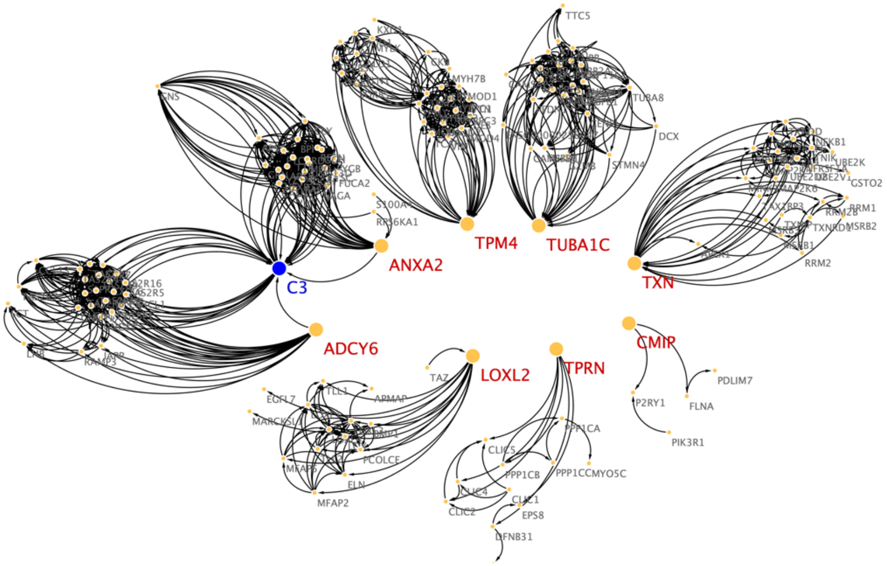

Figure S1. Protein-Protein network of TXN, ANXA2, TPM4, LOXL2, TPRN, ADCY6, TUBA1C and CMIP based on the STRING website. Orange is 8 key gene, blue is the connecting *ADCY6* and *ANXA2*.
